# Children’s moderate-to-vigorous physical activity on weekdays versus weekend days: a multi-country analysis

**DOI:** 10.1186/s12966-021-01095-x

**Published:** 2021-02-10

**Authors:** Keith Brazendale, Michael W. Beets, Bridget Armstrong, R. Glenn Weaver, Ethan T. Hunt, Russell R. Pate, Timothy A. Brusseau, Amy M. Bohnert, Timothy Olds, Rafael M. Tassitano, Maria Cecilia M. Tenorio, Jeanette Garcia, Lars B. Andersen, Rachel Davey, Pedro C. Hallal, Russell Jago, Elin Kolle, Susi Kriemler, Peter L. Kristensen, Soyang Kwon, Jardena J. Puder, Jo Salmon, Luis B. Sardinha, Esther M. F. van Sluijs, on behalf of the International Children’s Accelerometry Database (ICAD) Collaborators, S. Anderssen, G. Cardon, R. Davey, P. Hallal, K. F. Janz, S. Kriemler, N. Møller, K. Northstone, A. Page, R. Pate, J. J. Puder, J. Reilly, J. Salmon, L. B. Sardinha, E. M. F. van Sluijs

**Affiliations:** 1 Department of Health Sciences, College of Health Professions and Sciences, University of Central Florida, 4364 Scorpius Street, Orlando, FL 32816 USA; 2 Department of Exercise Science, Arnold School of Public Health, University of South Carolina, Columbia, SC USA; 3 Department of Health & Kinesiology, College of Health, University of Utah, Salt Lake City, UT USA; 4 Department of Psychology, Loyola University Chicago, College of Arts and Sciences, Chicago, IL USA; 5 Alliance for Research in Exercise, Nutrition and Activity (ARENA), University of South Australia, Adelaide, Australia; 6 Department of Physical Education, Federal Rural University of Pernambuco, Recife, PE Brazil; 7 Department of Teacher Education and Sport, Western Norwegian University of Applied Sciences, Sogndal, Norway; 8 Health Research Institute, University of Canberra, Canberra, Australia; 9 Postgraduate Program in Epidemiology, Federal University of Pelotas, Pelotas, Brazil; 10 Centre for Exercise, Nutrition and Health Sciences, University of Bristol, Bristol, UK; 11 Norwegian School of Sport Sciences, Oslo, Norway; 12 Epidemiology, Biostatistics and Prevention Institute, University of Zürich, Zürich, Switzerland; 13 University of Southern Denmark, Odense, Denmark; 14 Stanley Manne Children’s Research Institute, Ann & Robert H. Lurie Children’s Hospital of Chicago, Chicago, USA; 15 Service of Obstetrics, Centre Hospitalier Universitaire Vaudois, University of Lausanne, Lausanne, Switzerland; 16 Institute for Physical Activity and Nutrition, School of Exercise and Nutrition Sciences, ZDeakin University, Geelong, Australia; 17 Exercise and Health Laboratory, CIPER, Faculty of Human Movement, Universidade de Lisboa, Lisbon, Portugal; 18 MRC Epidemiology Unit & Centre for Diet and Activity Research, University of Cambridge, Cambridge, UK

**Keywords:** Children, Physical activity, Accelerometer, Weekday, Weekend, Structure

## Abstract

**Purpose:**

The Structured Days Hypothesis (SDH) posits that children’s behaviors associated with obesity – such as physical activity – are more favorable on days that contain more ‘structure’ (i.e., a pre-planned, segmented, and adult-supervised environment) such as school weekdays, compared to days with less structure, such as weekend days. The purpose of this study was to compare children’s moderate-to-vigorous physical activity (MVPA) levels on weekdays versus weekend days using a large, multi-country, accelerometer-measured physical activity dataset.

**Methods:**

Data were received from the International Children’s Accelerometer Database (ICAD) July 2019. The ICAD inclusion criteria for a valid day of wear, only non-intervention data (e.g., baseline intervention data), children with at least 1 weekday and 1 weekend day, and ICAD studies with data collected exclusively during school months, were included for analyses. Mixed effects models accounting for the nested nature of the data (i.e., days within children) assessed MVPA minutes per day (min/day MVPA) differences between weekdays and weekend days by region/country, adjusted for age, sex, and total wear time. Separate meta-analytical models explored differences by age and country/region for sex and child weight-status.

**Results/findings:**

Valid data from 15 studies representing 5794 children (61% female, 10.7 ± 2.1 yrs., 24% with overweight/obesity) and 35,263 days of valid accelerometer data from 5 distinct countries/regions were used. Boys and girls accumulated 12.6 min/day (95% CI: 9.0, 16.2) and 9.4 min/day (95% CI: 7.2, 11.6) more MVPA on weekdays versus weekend days, respectively. Children from mainland Europe had the largest differences (17.1 min/day more MVPA on weekdays versus weekend days, 95% CI: 15.3, 19.0) compared to the other countries/regions. Children who were classified as overweight/obese or normal weight/underweight accumulated 9.5 min/day (95% CI: 6.9, 12.2) and 10.9 min/day (95% CI: 8.3, 13.5) of additional MVPA on weekdays versus weekend days, respectively.

**Conclusions:**

Children from multiple countries/regions accumulated significantly more MVPA on weekdays versus weekend days during school months. This finding aligns with the SDH and warrants future intervention studies to prioritize less-structured days, such as weekend days, and to consider providing opportunities for all children to access additional opportunities to be active.

## Background

The World Health Organization recommends all children and adolescents (5–17 years) achieve 60 min per day of moderate-to-vigorous physical activity (MVPA) for health benefit [1]. Device-measured international estimates of children’s MVPA suggests that no more than 45% of children meet the daily recommendation [2, 3]. The majority of research aimed at improving children’s daily physical activity levels have taken place in the school setting [4], with school-based intervention efforts falling short of making a positive impact on children’s physical activity across the full day [5].

In contrast to the wealth of evidence on children’s school-based physical activity, there are fewer studies on children’s physical activity levels during times when they are not in school, such as during summer months. This is of particular importance as recent longitudinal evidence has shown that during summer vacation children exhibit accelerated weight-gain in comparison to school months [6, 7]. One possible reason for this may be due to the limited ‘*structure’* of the day during the summer. The ‘Structured Days Hypothesis’ (SDH) was developed to help understand differences in children’s obesogenic behaviors during school versus summer months [8]. The SDH posits that a ‘structured day’ is a pre-planned, segmented, and adult supervised compulsory environment that a child is exposed to on any given day. The consistent presence of routine, and/or regulation within the day positively shapes the obesogenic behaviors of children and adolescents (i.e., increased daily physical activity) [8]. The most common example of a ‘structured day’ is a day when a child or adolescent attends school, but other examples exist in the form of daycare, day camps or programs. The key underlying distinction of a ‘structured day’ is that the whole structure of the day is shaped or influenced by the very presence of the school/camp/program’s consistent start and end times, and by the various compulsory components presented to attending children and adolescents. For example, on a ‘structured day’ such as a school weekday, from the moment a child wakes up to the time they go to bed, elements of routine, regulation, and adult-supervised compulsory components exist. This presents several *intentional* (e.g., free play time before or after school hours, school recess, physical education, classroom physical activity breaks) and *unintentional* (e.g., child has consistent earlier wake time so more time in day to be physically active, active transport to and from school, segmented components of school day elicit transitions between activities) opportunities for the child to be active that exist inside and outside of school operating hours. A ‘structured day’ provides these different types of opportunities, both intentional and unintentional, and in a relatively unavoidable and involuntary nature.

Since its publication in 2017, the SDH has informed several studies that have purposefully compared obesogenic behaviors during structured versus less-structured times, such as summer versus school months [9, 10], summer camp/program days versus non camp/program days [11–13], and school-days versus non-school days [14–16]. Together, these studies align with the notion that children’s obesogenic behaviors are more favorable on structured versus less-structured days, yet are limited by small sample sizes and specific population demographics, making it challenging to generalize findings.

In the absence of robust evidence, exploring weekday versus weekend day estimates of obesogenic behaviors could be viewed as the ‘*next best’* example one could draw from to compare a structured (e.g., weekdays) versus a less-structured day (e.g., weekend days). Previously, the SDH explored studies comparing MVPA estimates of elementary school-aged children on weekday versus weekend days, concluding that ~ 80% of studies (*n* = 91) were in favor of the hypothesis (i.e., MVPA greater on weekdays) [8]. However, the included studies varied in method of physical activity assessment (device-measured vs. self-report) and focused solely on elementary school-aged children (5–11 years old). A separate systematic review and meta-analysis [17] acknowledged that school-aged children’s MVPA was greater on weekdays versus weekend days, but noted outcome measure (e.g., studies reporting MVPA minutes versus accelerometer counts per minute) can influence conclusions thus making it difficult to draw comparisons across studies. Moreover, recent studies have reported weekday versus weekend day accelerometer-derived MVPA estimates [18–24]. Collectively, the majority of these studies report higher MVPA estimates on weekdays compared to weekend days, however these data have been limited by small sample sizes [20, 23], examination of specific age ranges [19, 22, 24] and populations [21, 23], and the time these data were collected (i.e., not exclusively during school months) [18]. Thus, drawing conclusions in the context of the SDH is, to some extent, limited.

The purpose of this study was to explore whether MVPA differences exist between weekdays and weekend days using a large, international, accelerometer-measured physical activity dataset of children and youth (age 6 to 18 years), exclusively focusing on school month data. By examining school month data only, the authors can establish a clear comparison of ‘structured days’ (school weekdays) versus ‘less-structured day’ (weekend days). The authors hypothesize that children will exhibit higher levels of MVPA on weekdays versus weekend days, showing support toward the SDH.

## Methods

### Study design and sample

This study used secondary data provided by the International Children’s Accelerometry Database (ICAD), received July 2019. ICAD is a database of pooled data on accelerometer-assessed physical activity from 21 studies in children and adolescents worldwide. Detailed information on the methods of the ICAD project can be found elsewhere [25]. In short, raw accelerometery files were obtained from cross-sectional, longitudinal, and intervention studies, that measured physical activity with waist-worn Actigraph accelerometers (*Models*; *7164*, *71,256*, *GT1M1*, Actigraph LLC, Pensacola, FL) on children and youth from 3 to 18 years old. All participants and/or their legal guardian provided written informed consent and local ethical committees from each contributing ICAD study approved individual study protocols. Prior to sharing data, data-sharing agreements were established between contributing studies and MRC Epidemiology Unit, University of Cambridge, UK. This secondary data analysis reports within the guidelines of the STROBE Statement.

Not all ICAD studies were eligible for data analyses. In addition to the ICAD inclusion criteria [25], a study was included for analyses in the present study if the additional criteria could be met: data were only collected during a school month (i.e., all data that included holiday/school break/summer months excluded as month of data collection was not available); data represented children between the ages of 6–18 years; and, non-intervention physical activity data was obtainable (e.g., baseline data for intervention and longitudinal studies). For the present analysis, 15 studies met the inclusion criteria outlined above (out of 21 eligible studies), and data from the 15 studies were recoded to represent the following 5 countries/regions; the United Kingdom (*n* = 3), mainland Europe (*n* = 7), United States of America (*n* = 2), Australia (n = 2) and Brazil (*n* = 1). Eight of the contributing 15 studies were longitudinal or intervention studies, therefore, baseline values were used.

### Data preparation

Child accelerometry observation days were removed if they did not meet a valid day of wear; defined as ≥600 min/day [26], and if a child did not have at least one valid weekday and weekend day. As per the ICAD, studies contributing data files which used an epoch < 60 s were reintegrated up to 60 s for analysis [25]. The selection of an appropriate accelerometer cut-point for MVPA is an important issue in studies employing accelerometry to measure physical activity of children and youth [27]. Previous studies have shown that for children and adolescents (ages 5 to 15 years) the best prediction, specificity, and sensitivity [28], from a range of widely-used accelerometer-cut points was provided by Evenson’s cut-points [29], despite the fact that Evenson’s cut-points were originally validated in a group of younger children (5 to 9 year-olds). Thus, the present study incorporated ≥2296 cpm to define children’s MVPA. In addition to MVPA, children’s age, sex, height, and weight were available from all included studies. Children’s height and weight were transformed into body mass index (BMI) values by taking the weight in kilograms and dividing it by the square of height in meters (kg/m^2^). BMI categories (e.g., normal weight, with overweight, with obesity etc.) were established using age- and sex-specific growth curves endorsed by the World Health Organization [30]. Children with a BMI ≥85th percentile (for age and sex) were classified as ‘overweight/obese’ (1) and compared to the rest of the sample, labeled ‘normal weight/underweight’ (<85th percentile) (0).

### Data analyses

Mixed effects models accounting for the nested nature of the data (i.e., multiple observation days per child) assessed overall MVPA differences between weekday and weekend days, and by country/region. These models adjusted for age, sex, and total daily wear time. Random effects meta-analytical regression models, weighted by sample size at the study-level, were computed to explore MVPA differences between weekday and weekend days of individual studies by age (6 to 16 years), sex (boys vs. girls), and weight-status (overweight/obese vs. normal weight/underweight). Meta-analyses excluded data from a given study if there were 5 or fewer children for a single age year or BMI category. Meta-analyses were performed by subgroups of age and location, with data pooled across studies using the DerSimonian–Laird method. Because all studies measured the outcome of interest on the same scale (minutes per day of MVPA), the raw (unstandardized) mean difference was computed for the effect size. Heterogeneity was determined by the I^2^ statistics to assess the variability in effect estimates [31]. Forest plots were generated to present overall effects, and effects by region. Outcomes were expressed in MVPA minutes accumulated per day (min/day of MVPA). A sensitivity analysis was performed to explore MVPA differences on an ‘average’ weekday versus an ‘average’ weekend day for each child (i.e., MVPA estimates are averaged for each child so they contribute 1 weekday and 1 weekend estimate to the analysis), and raw min/day of MVPA estimates were calculated to explore day-to-day patterns. All analyses were conducted in Stata (version 16.1, College Station, TX, USA).

## Results

**Fig. 1:**
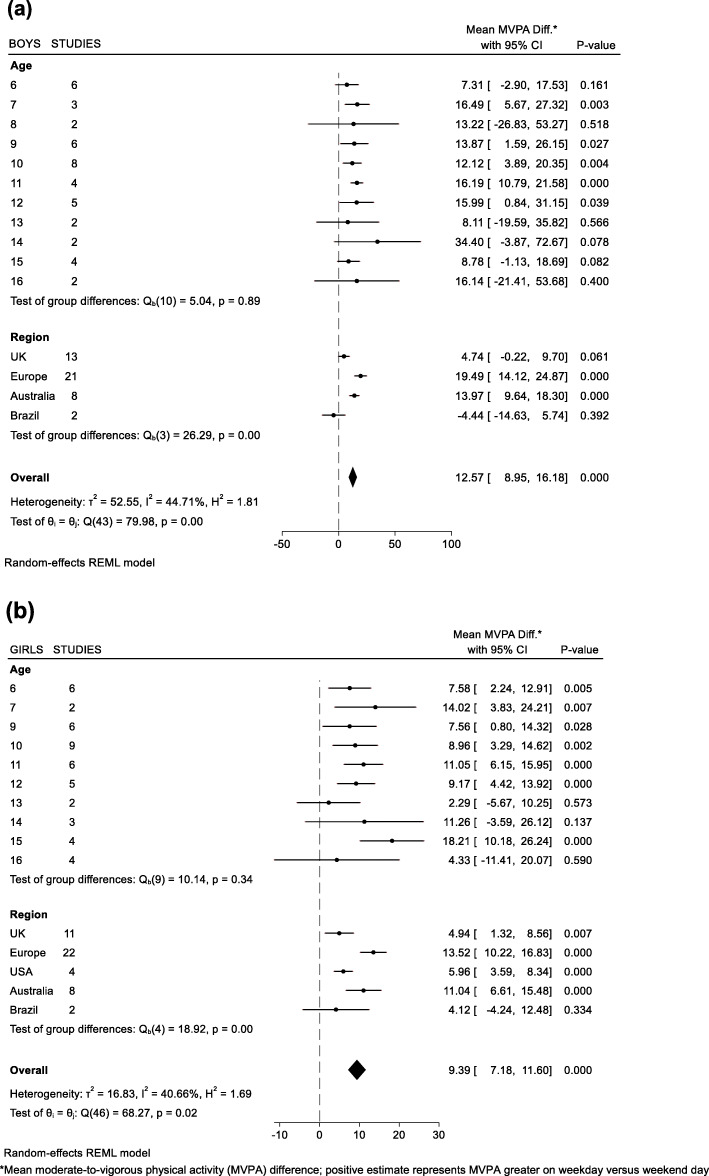
Mean differences in MVPA on weekdays versus weekend days of (**a**) Boys and (**b**) Girls

**Fig. 2:**
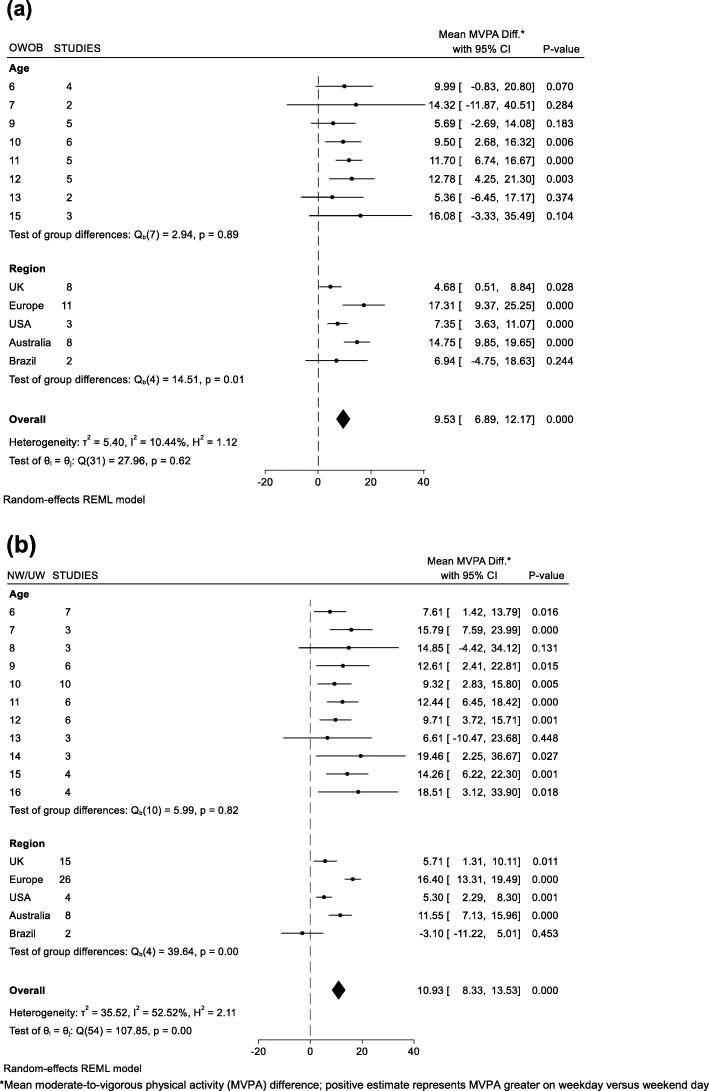
Mean differences in MVPA on weekdays versus weekend days of children who are (**a**) Overweight/Obese (OWOB) and (**b**) Normal weight/Underweight (NW/UW)

**Fig. 3:**
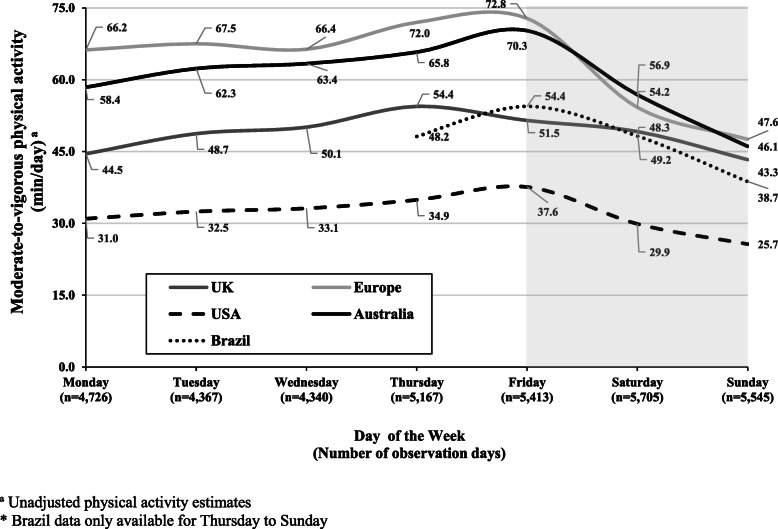
Children’s mean moderate-to-vigorous physical activity by day of the week. Shaded area represents weekend

A summary of child and country/region characteristics are presented in Table 1. A total of 5794 children (mean age 10.7 yrs., 61% girls, 24% with Overweight/Obesity) with 35,263 valid days of accelerometry. Mean total wear time on weekdays was 800.5 min/day compared to 805 min/day on weekend days. Mean (±SD) valid weekday and weekend day accelerometer days were 3.7 (±1.3) and 1.8 (±0.4) per child, respectively. Results from the sensitivity analysis (average weekday versus average weekend day) showed children accumulated 55.1 min/day of MVPA on weekdays compared to 45.7 min/day of MVPA on weekend days. Low-to-moderate heterogeneity [31] was observed across meta-analyses (I^2^ range: ~ 10–50%) as indicated on the forest plots for min/day of MVPA differences by sex and BMI category (Figs. 1 and 2). Mixed effects models revealed age, the interaction between age and sex, and the interaction between age and BMI were not significant predictors of differences between weekday and weekend day MVPA. Boys accumulated an additional 12.6 min/day of MVPA (95% CI: 9.0, 16.2) on weekdays versus weekend days, with boys in European countries contributing the largest differences in min/day of MVPA on weekdays versus weekend days (+ 19.5 min/day of MVPA, 95% CI: 14.1, 24.9) (Fig. 1a). Girls accumulated an additional 9.4 min/day of MVPA (95% CI: 7.2, 11.6) on weekdays versus weekend days, with girls in European countries contributing the largest differences in min/day of MVPA on weekdays versus weekend days (+ 13.5 min/day of MVPA, 95% CI: 14.1, 24.9) (Fig. 1b). Children who were classified as overweight/obese or normal weight/underweight accumulated an additional 9.5 min/day of MVPA (95% CI: 6.9, 12.2) and 10.9 min/day of MVPA (95% CI: 8.3, 13.5) on weekdays versus weekend days, respectively (Fig. 2). Children from European countries who were either overweight/obese (+ 17.1 min/day of MVPA, 95% CI: 9.4, 25.3) or normal weight/underweight (+ 16.4 min/day of MVPA, 95% CI: 13.3, 19.5) displayed the highest min/day of MVPA differences between weekdays versus weekend days when results were analyzed by weight-status. We did not find significant weekday versus weekend day differences in MVPA among Brazilian children in this study. Figure 3 displays unadjusted MVPA estimates for Monday to Sunday for each country/region.

**Table 1:** Study sample characteristics stratified by country/region

Country/ Region (number of studies)	Days (n)	Children (n)	Age														Girls (%)	OWOB (%)	Moderate-to-Vigorous Physical Activity (MVPA) Minutes/Day							MVPA Difference WD vs WE* (95%CI)
			6	7	8	9	10	11	12	13	14	15	16	Mean	±SD	Range			All Days		Weekdays (WD)			Weekend Days (WE)		
																			Mean	±SD	Mean	±SD		Mean	±SD	
**All** (15)	35,263	5794	•	•	•	•	•	•	•	•	•	•	•	10.7	2	(6–16)	61	24	51.6	26	54.3	36		45.7	39	**8.9, (8.2,9.7)**
**Europe** (7)	7369	1471	•	•	•	•	•	•	•	•	•	•	•	9.8	3	(6–16)	51	13	62.3	43	69.7	43		50.9	41	**17.1, (15.3,19.0)**
**UK** (3)	12,202	1852	•	•	•	•	•	•	•	•	•	•	•	10.6	1	(6–16)	54	23	48.8	34	50.0	31		46.3	40	**3.6, (2.3,4.9)**
**USA** (2)	5833	920	•				•	•	•	•	•			11.9	1	(6–14)	99	31	32.1	25	34.0	24		27.8	28	**6.1, (4.7,7.5)**
**Australia** (2)	8737	1300	•	•	•	•	•	•	•	•				10.6	2	(6–13)	54	27	60.4	36	64.1	35		51.5	38	**12.6, (11.0,14.2)**
**Brazil** (1)	1122	251							•	•				13.3	0	(6–14)	48	26	43.3	39	43.0	38		43.5	40	−0.7, (−5.3,3.7)

*OWOB* overweight or obese, *±SD* standard deviation

*Model-derived estimates adjusted for age, sex, and total wear time; Bolded values indicate statistically significant difference between WD versus WE (*p* < 0.05)

## Discussion

The purpose of this study was to compare children’s device-measured physical activity estimates on weekdays versus weekend days. Results from this analysis show that boys and girls accumulate more MVPA on weekdays compared to weekend days. MVPA differences between weekdays and weekend days were observed across most regions/countries but were most pronounced in European countries and Australia. These findings provide evidence aligning with the SDH, specifically showing that weekdays during school months can have a positive impact on boys’ and girls’ health-enhancing levels of physical activity.

In the current analyses, boys and girls accumulated an additional 12 min/day of MVPA and 9 min/day of MVPA, respectively, on weekdays compared to weekend days, which falls within the range of recent individual studies investigating accelerometer-derived weekday versus weekend differences in MVPA (+ 4 to + 25 min/day of MVPA on weekdays) [18–24]. Interestingly, no patterns emerged for differences between weekday and weekend day MVPA with age. It is well understood that physical activity declines as children transition from childhood to adolescence [32–34], however, longitudinal research has found that with increasing age, MVPA declines on weekend days more so than on weekdays [32]. This highlights an important consideration to promote weekend physical activity, especially as children grow older, and overall physical activity levels decline. Within the context of the SDH, adult control on how children spend their time undoubtedly changes as children grow older [35], however, school weekdays – and the accompanying opportunities to remain engaged in extra-curricular activities – are commonplace from childhood into adolescence. During weekend days, when there is conceivably less structure, children have more autonomy over their time and may choose less healthful pursuits (e.g., excessive screen time use) [8]. This notion aligns with international literature on screen time which reports consistently higher daily screen time estimates on weekend days compared to weekdays [36–39], and when presented with a choice, children could be opting for the less-healthful alternatives (e.g., choosing sedentary pursuits over being active) [40, 41]. In addition, the SDH draws from concepts found in the ‘filled-time perspective’ which is based on the principal that time filled with favorable activities cannot be filled with unfavorable activities [42]. A ‘typical’ school day, in and of itself, fills a considerable chunk of time for all children that can significantly reduce engagement in less favorable activities. Although measures exist that can capture and quantify ‘how’ children spend their time [43], further studies incorporating these measures on both structured and less-structured days are warranted.

Notably, the majority of intervention efforts have focused on children during weekdays during the school year, where little-to-no impact has been made on children’s MVPA [4, 5]. A recent meta-analysis of 17 randomized controlled trials spanning North/South America, Europe and Australia, reported null effects for influencing accelerometer-assessed weekday MVPA [5]. From a pragmatic standpoint, it makes sense to focus intervention efforts and resources on weekdays during school (e.g., recruiting a whole school, access to associated school-based programs/personnel/resources etc.), as children spend a large portion of their time in-and-around the school environment. However, given the lackluster outcomes of school-based interventions and in light of results from the current study, it may be more effective to focus intervention efforts to weekend days [17]. A weekday during school months may be at ‘*capacity’* in terms of room for additional opportunities for children to be active. On weekend days, children are not guaranteed consistency to their days like they are during a ‘structured’ school day, therefore, less intentional and unintentional physical activity opportunities may be present, thus, leaving room for more unfavorable activities (e.g., screen time/sedentary pursuits). Therefore, weekend days might be a time where intervention efforts could be most beneficial to improving MVPA.

Across all countries/regions examined in this study, both boys’ and girls’ MVPA was greater on weekdays compared to weekend days, except for children in the contributing Brazil study, and boys in the pooled UK-based studies. These findings are largely consistent with the body of literature that has presented MVPA estimates of children on weekdays and weekend days from these countries/regions [23, 32, 44–46]. Of more interest, children from mainland Europe and Australia, by comparison to the others, accumulate more MVPA on both weekdays and weekend days, however, the magnitude of the differences observed between weekdays and weekend days from European and Australian children are noticeably higher than other regions/countries; and the potential reasons for this are not entirely clear. One possible explanation may be that the collective components of the weekday during school months (in Europe and Australia) are providing more intentional and unintentional physical activity opportunities for European and Australian children, whether it be active transport to and from school, increased access to before or after school programs/clubs [47], or more dedicated time in the school curriculum (e.g., duration and frequency) for the accumulation of MVPA through recess, classroom breaks, and physical education [48]. This may not be the case for children from the USA, UK, and Brazil. For example, in the USA fewer public schools have adopted all components of the comprehensive school physical activity program, which calls for multiple school-related environments (e.g., before and after school time) to provide comprehensive and consistent physical activity opportunities for children [49]. Perhaps more tellingly, the recent Global Matrix (3.0) Physical Activity Report Card [50] analyzed 49 countries on several physical activity indicators and gave overall lower grades to the USA, UK, and Brazil in comparison to Australia and European Countries in this study on the indicators ‘*Community and Environment*’, ‘*School’*, and ‘*Organized Sport’*. This suggests that the necessary components, support, and environmental infrastructure that could impact children’s MVPA on both weekdays (i.e., school environment) and weekend days (i.e., organized sport and community-based activity opportunities) is not adequate in these regions.

There are other potential individual and environmental mechanisms that may explain the observed differences among countries/regions. These current data were collected during school months, however, the countries/regions in this sample vary drastically in terms of weather conditions. Previous research using ICAD data reported that children from Australia and northern European countries have higher activity levels given the weather conditions they experience compared to those in Western Europe and USA [51]. The authors concluded that typically, relationships between weather (e.g., temperature, visibility, wind speed, precipitation) and physical activity levels of children are linear, however, for extreme temperatures over 20 °C physical activity levels begin to decrease. Within the current study, the availability of indoor temperature-controlled environments conducive towards physical activity in extreme weather climates [52] may or may not have been available for children, and, thus, could influence the MVPA estimates presented herein. Future research of children’s physical activity levels during different seasons and over time, should consider weather conditions as a determinant, particularly in younger children (e.g., pre-school and primary-school age) [51]. On the individual level, the influence of other behaviors such as sleep patterns (e.g., early to bed/early to rise), which favor MVPA on weekdays [53], and the penetration of more sedentary pursuits (e.g., screen time) on weekend days where time requirements of family members are less stable in comparison to the ‘5-day work week’ [54], could be contributing to some of the differences observed between weekday and weekend day MVPA.

As mentioned previously, no differences were observed between weekday and weekend MVPA of children from Brazil. This is in opposition to a more recent study of Brazilian children where an additional ~ 7 min of MVPA was accumulated on weekdays versus weekend days [39]. One reason for this may be because at the time these data were collected (2006–2007) “Integral Education” was not commonplace in Brazilian public schools [55, 56]. Prior to the implementation of integral education (pre-2008), children who attended public schools in Brazil could attend school for a self-selected time of 4 to 5 h per day (e.g., 7:30 am – 12 pm, or 1:00 pm – 5:00 pm), and opportunities to attend school-based extra-curricular programming was limited and not accessible to children from low-income households. With the introduction of the integral education initiative, Brazilian public schools are tasked with offering 35–45 h per/week of schooling and extra-curricular resources to all children and families [56]. Thus, within the confines of the SDH, it is conceivable that the actual ‘dose’ of structure afforded to Brazilian children on weekdays in the analyzed sample was less than that of both children from the other contributing studies in this analyses, and subsequent Brazilian studies that have explored physical activity patterns of children from Brazil since integral education adoption. Lastly, physical spaces (e.g., indoor gymnasiums) for children to be active are not as commonplace in Brazilian public schools compared to private schools in Brazil, which could be another reason for the null findings in the present analyses [57].

Studies have reported vast differences in accelerometer-derived MVPA on specific days of the week, such as boys and girls achieving an additional 26.9 min/day of MVPA and 16.9 min/day, of MVPA, respectively, on a Friday compared to a Sunday [54]. In that study, the authors highlighted that a reoccurring weekly pattern was emerging in children from 8 years of age all the way through to age 12, where physical activity levels increased throughout the week leading up to Friday after which, a drop off in MVPA was observed. The authors speculated that socio-cultural influences of the working week common to ‘modern society’, whereby a weekly cycle is dictated by 5 days of school or work for the family, followed by 2 ‘rest/free’ days could be contributing to these differences in weekday and weekend day physical activity. These patterns emerged in the current data displayed in Fig. 3 showing children from different countries/regions steadily increasing activity levels from Monday to Friday before tailing off over the weekend, with Sunday producing the lowest MVPA estimate. This brings into line the SDH and demonstrates the positive impact the school week can have on all children’s physical activity levels. Weekdays during school months somewhat obligate children to engage in this consistent routine of pre-planned, segmented and adult-supervised structured days that build ‘momentum’ leading up to the weekend, where children and families can choose to go ‘at their own pace’. Yet, these current data also highlight how unique both Saturday and Sunday are, with Sunday producing the lowest MVPA estimates in comparison to other days across all countries/regions, with noticeably lower estimates in comparison to Saturday. Generally, Saturday’s are viewed as the day when intentional physical activity opportunities exist in the form of sport or activity-based programs, whereas Sunday’s could be viewed as the day reserved for rest/more sedentary pursuits, religious practices, family time, and/or homework tasks [58]; activities that are not necessarily conducive towards accumulating MVPA. Understanding such physical activity patterns and nuances may assist public health practitioners in designing more specific interventions to increase physical activity levels in children.

Data have shown that schools can provide a “homogenizing regime” [59] for children’s health or an “equalizing effect” [60] on children’s physical and cognitive abilities, regardless of background or socioeconomic status. This is evident within the current findings that show a similar magnitude of difference for both girls, and children with overweight or obesity; subgroups of children that typically display lower levels of MVPA in field-based accelerometer studies, in comparison to their counterparts [61]. As highlighted by the SDH, the school weekday can provide multiple opportunities for intentional and unintentional physical activity for every child, essentially breaking up long periods of sedentary time. Such opportunities and occurrences are not guaranteed during weekend days, and studies support this notion reporting that the frequency of long periods of time (i.e., ‘bouts’) spent in MVPA is lower on weekend days compared to weekdays [18], suggesting a higher frequency of uninterrupted MVPA occurrences on weekdays. Future intervention research exploring weekend day physical activity may consider targeting bout frequency as a possible point of intervention.

Traditionally, physical activity opportunities during less-structured days (e.g., weekend days, summer days) exist in the form of organized sport participation, day camps, and programs. For example, the USA and Australia have reported relatively high participation rates among children (60–85%) in organized sport, with Europe showing lower estimates in comparison [62]. Further, accelerometer-derived MVPA estimates for children who attend general programming – such as a summer day camp – are notably high [63]. However, there is a cost to attending these existing physical activity opportunities, meaning children from low-income households are less likely to attend; a trend that is recognized regardless of region [59, 62, 64, 65]. One promising strategy to enhance children’s MVPA during less-structured days is to make existing community-based facilities and resources that are accessible during weekdays or school months, available during weekend days or summer (e.g., opening school grounds/space for free-play or structured programming) [19, 66]. Another strategy is to consider demand-side financing [67, 68], where children/families would be provided financial assistance in the form of a voucher to attend locally operated programs. Collectively, such strategies would help address previous barriers to weekend participation in organized physical activity (e.g., payment of club/program fees, transportation to facilities outside of the community) [69]. Other studies have noted that parental support towards physical activity (e.g., verbal encouragement, positive feedback, availability of parental participation) [45, 70] is an important determinant of children’s physical activity levels, and, thus, adopting intervention strategies – such as those mentioned above – that specifically target times when children are consistently less active (i.e., weekend days) need to consider contextual information such as the role of the family/parent and the home environment.

There are several strengths to the current study. First, the findings presented herein represent device-measured estimates of MVPA. Second these data represent a large, multi-national sample of children covering a wide range of ages (6–16 years). Third, by considering only school months data (i.e., school weekdays) for this study, a typical ‘structured day’ for children can be compared to weekend days, considered a less-structured day, for a strong examination of the SDH. There are limitations to this study that must be acknowledged. Accelerometer-derived estimates of MVPA can vary drastically based on cutpoint selection [27], however the authors selected a cutpoint that offered the best prediction, specificity, and sensitivity that could handle a range of ages [28]. The authors also note that there are other factors (e.g., days of monitoring, study site protocols) that can impact MVPA estimates when making between-study and international comparisons [71]. Seasonality was not addressed in the current analyses, and can influence weekday versus weekend day differences of physical activity across various countries/regions [52, 72]. In addition, socioeconomic status was not considered in the current analyses due to the missingness (~ 35%) of a socioeconomic indicator (e.g., parental education) for certain children.

## Conclusions

In total, the data presented herein demonstrate that weekdays during school months can provide children with significant and meaningful additional minutes of MVPA compared to weekend days. This finding is consistent with the premise of the SDH. Public health practitioners and individuals responsible for the care of children need to consider appropriate access to opportunities and promoting physical activity during days when structure is not inherently present for all children, like it is during school weekdays [17, 19, 45, 54, 73]. Future studies should look to adopt longer wear-time protocols outside of the traditional 7-day accelerometer protocol – whereby 3 weekdays and 1 weekend day is deemed sufficient – to gain more insight of children’s long-term physical activity patterns during different periods of structured versus less-structured days (e.g., 1 ‘school’ month versus 1 ‘summer’ month) [74]. In addition, future studies may want to explore other intensities of physical activity (e.g., light, vigorous) during weekday versus weekend days, and for 24-h wear protocols, the interrelation of sleep and activity using compositional data analysis. Finally, although the present findings align with the SDH, the authors recognize there are other individual and environmental-level factors that exist, such as differential sleep patterns on weekdays and weekend days [75], that could explain differences in MVPA estimates.

To conclude, children’s device-measured MVPA is greater on weekdays compared to weekend days during school months. Researchers, public health practitioners, and policy makers need to consider prioritizing days when children are not afforded the consistency and routine of the school weekday – such as weekend days – to increase physical activity among children and youth.

## Data Availability

The data that support the findings of this study are available from MRC Epidemiology Unit, Cambridge. But restrictions apply to the availability of these data, which were used under license for the current study, and so are not publicly available. Data are however available from the authors upon reasonable request and with permission of MRC Epidemiology Unit, Cambridge.

## Abbreviations

SDH: Structured days hypothesis
MVPA: Moderate-to-vigorous physical activity
ICAD: International Children’s Accelerometry Database
95%CI: 95% Confidence interval
CPM: Counts per minute
BMI: Body mass index
SD: Standard deviation
UK: United Kingdom
USA: United States of America
